# SIRT6 stabilizes DNA-dependent Protein Kinase at chromatin for DNA double-strand break repair

**DOI:** 10.18632/aging.100011

**Published:** 2009-01-15

**Authors:** Ronald A. McCord, Eriko Michishita, Tao Hong, Elisabeth Berber, Lisa D. Boxer, Rika Kusumoto, Shenheng Guan, Xiaobing Shi, Or Gozani, Alma L. Burlingame, Vilhelm A. Bohr, Katrin F. Chua

**Affiliations:** ^1^ Department of Medicine, Stanford University School of Medicine, Stanford, CA 94305, USA; ^2^ Geriatric Research, Education and Clinical Center, VA Palo Alto Health Care System, Palo Alto, CA 94304, USA; ^3^ Department of Molecular Gerontology, National Institute on Aging, NIH, Baltimore, MD 21224, USA; ^4^ Department of Pharmaceutical Chemistry and Mass Spectrometry Facility, University of California, San Francisco, CA 94143, USA; ^5^ Department of Biological Sciences, Stanford University, Stanford, CA 94305, USA; ^6^ These authors contributed independently to this work

**Keywords:** Sir2, SIRT6, genomic stability, DNA repair, DNA damage, aging

## Abstract

**The Sir2 chromatin regulatory factor links maintenance of genomic stability to life span extension in yeast.  The mammalian Sir2 family member SIRT6 has been proposed to have analogous functions, because SIRT6-deficiency leads to shortened life span and an aging-like degenerative phenotype in mice, and SIRT6 knockout cells exhibit genomic instability and DNA damage hypersensitivity.  However, the molecular mechanisms underlying these defects are not fully understood.  Here, we show that SIRT6 forms a macromolecular complex with the DNA double-strand break (DSB) repair factor DNA-PK (DNA-dependent protein kinase) and promotes DNA DSB repair.  In response to DSBs, SIRT6 associates dynamically with chromatin and is necessary for an acute decrease in global cellular acetylation levels on histone H3 Lysine 9.  Moreover, SIRT6 is required for mobilization of the DNA-PK catalytic subunit (DNA-PKcs) to chromatin in response to DNA damage and stabilizes DNA-PKcs at chromatin adjacent to an induced site-specific DSB.  Abrogation of these SIRT6 activities leads to impaired resolution of DSBs.  Together, these findings elucidate a mechanism whereby regulation of dynamic interaction of a DNA repair factor with chromatin impacts on the efficiency of repair, and establish a link between chromatin regulation, DNA repair, and a mammalian Sir2 factor.**

## Introduction

The Silent Information Regulator-2 gene (Sir2) encodes an NAD-dependent histone deacetylase that links regulation of chromatin, genomic stability, and life span in S. cerevisiae.  By  promoting  chromatin silencing,  Sir2 inhibits transcription at several genetic loci and represses recombination at ribosomal DNA (rDNA) repeats [1-3].  Yeast with mutations in Sir2 have increased genomic instability in the context of rDNA recombination, which in turn shortens replicative life span - a marker of reproductive aging in this organism [4]. Conversely, extracopies of Sir2 that suppress rDNA recombination increase replicative life span [4].  These effects of Sir2 suggest paradigms in which genes that promote genome stabilization through chromatin modulation may be important contributors to regulation of organismal life span, aging, and age-related pathology.

Consistent with a conserved role for Sir2 factors in life span regulation, increased activity of Sir2 proteins in the multicellular organisms C. elegans and D. melanogaster also increases life span [5,6].  However, these Sir2 factors may operate through mechanisms that are independent of genome stabilization, and their physiologic molecular substrates are still unclear.  In mammals, there are seven Sir2 family members, SIRT1-SIRT7 [7,8].  The SIRTs have been of great interest as candidate regulators of mammalian life span and aging-related processes.  In this context, several mammalian SIRTs have functions that impact on aging-associated molecular pathways and disease [9,10].  However, initial studies of mammalian SIRTs linked these enzymes to biochemical targets and cellular functions that are distinct from those of S. cerevisiae Sir2.  For example, mammalian SIRT1 was first reported to deacetylate the p53 tumor suppressor protein [11,12]; only later was SIRT1 shown to have a physiologic role in histone deacetylation, chromatin regulation, and most recently, genome stabilization [13,14].  Other mammalian SIRTs (SIRT2-SIRT5) are reported to have cytoplasmic or mitochondrial substrates (though recent work suggests that sub-cellular shuttling might allow these enzymes to target histones as well) [9,10].  In addition, several studies had not detected histone deacetylase activity for the other nuclear SIRT proteins, SIRT6 and SIRT7 [15,16].  Thus, until recently, the extent to which the functional link of yeast Sir2 to chromatin and genome maintenance is evolutionarily conserved in mammals has been unclear.

The generation of mice deficient for the mammalian SIRT6 gene revealed a potential role for SIRT6 in linking regulation of life span, chromatin, and genomic stability [17].  In this context, SIRT6 deficiency in mice leads to dramatically shortened life span and acute degenerative phenotypes that overlap with pathologies of premature aging.  Moreover, SIRT6 knockout mouse cells have genomic instability and DNA damage hypersensitivity.  In biochemical fractionation assays, SIRT6 protein associates preferentially with a chromatin-enriched cellular fraction [17].  Together, these observations suggested that SIRT6 might couple chromatin regulation with DNA repair. However, a physiologic role for SIRT6 in such a process has not yet been demonstrated.

We recently discovered a molecular function for SIRT6 at chromatin.  We showed that SIRT6 deacetylates a specific histone residue, lysine 9 of histone H3 (H3K9), in the context of chromatin at telomeres [18].  SIRT6 thereby stabilizes the association with telomeres of the protein WRN, a DNA metabolic factor that is mutated in the human progeria Werner Syndrome.  In this context, depletion of SIRT6 in human cells leads to telomere dysfunction and genomic instability with end-to-end chromosomal fusions.  We also identified a second physiologic context for SIRT6 function as a histone H3K9 deacetylase [19].  Specifically, SIRT6 is recruited to the promoters of genes that have been activated by the NF-κB transcription factor, deacetylates H3K9 at these promoters to attenuate gene expression, and thereby limits NF-κB signaling.  Notably, hyperactive NF-κB signaling contributes significantly to the degenerative phenotypes and early death of SIRT6-deficient mice, because in an NF-κB-haploinsufficient genetic background, SIRT6-deficient mice have milder phenotypes and live much longer than mice with SIRT6-deficiency alone [19]. Thus, chromatin regulation by SIRT6 is important for proper telomere function and regulation of gene expression programs, and both these mechanisms of action may impact on genomic stability and aging.

Here, we report findings that further expand the known functions of SIRT6 and show that SIRT6 is required for efficient DNA DSB repair in the context of chromatin.  Biochemical analyses show that SIRT6 associates dynamically with chromatin in response to DNA damage, and stabilizes the DNA DSB repair factor, DNA-dependent protein kinase (DNA-PK), at DSBs.  We suggest that the modulation of DSB repair by SIRT6 in response to chronic DNA damage over life may contribute to the effects of SIRT6 on physiologic and pathologic processes in mammalian aging.

## Results

### SIRT6 interacts with the DNA DSB repair factor DNA-PK

**Figure 1.**
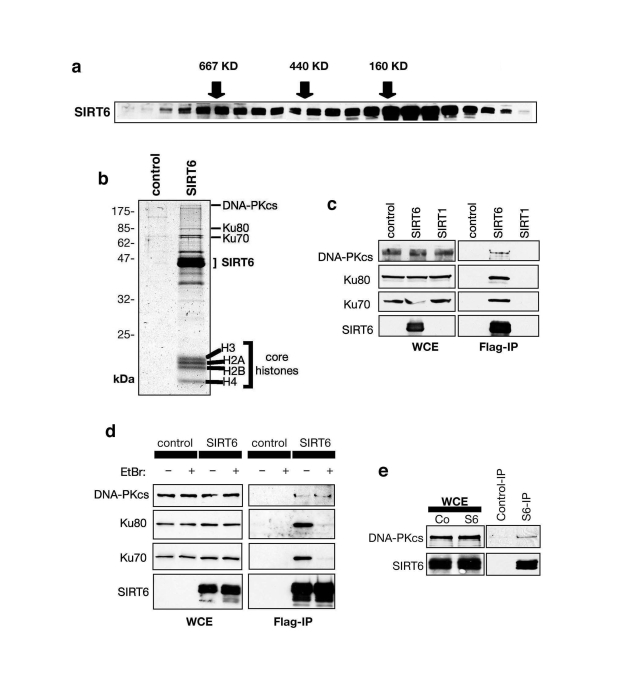
SIRT6 interacts with the DNA-PK DSB repair factor. (**A**) Endogenous SIRT6 protein associates with large molecular weight complexes.  HeLa cell nuclear extract (NE) was separated by gel-filtration and fractions subjected to Western analysis with SIRT6 antibody.  Fractions with molecular weight standards are indicated (arrows).  (**B**) Coomassie stain of proteins in Flag-SIRT6 or negative control IPs from 293T cells. The identities of proteins detected by mass spectrometry are indicated.  (**C**) SIRT6, but not SIRT1, associates with DNA-PK subunits.  Western analysis of SIRT6, SIRT1, and negative control IPs with the indicated antibodies.  WCE: whole cell extract.  (**D**) SIRT6 interaction with DNA-PKcs, but not Ku70 and Ku80, is resistant to ethidium bromide (EtBr).  Western analysis as in (**C**) except EtBr was added as indicated to disrupt DNA-mediated interactions.  (**E**) Endogenous interaction between SIRT6 and DNA-PKcs.  Western analysis of SIRT6-bound proteins in co-IPs from 293T cells.

To identify new molecular pathways of SIRT6 function, we employed a biochemical approach to identify SIRT6-interacting factors.  HeLa cell nuclear extracts were size-fractionated by gel filtration, and the presence of SIRT6 in individual fractions assessed by Western analysis.  Rather than fractionating at the expected size of a SIRT6 molecule (~40 KD), SIRT6 was detected in multiple peaks corresponding to large protein complexes ranging up to ~700 KD (Figure 1a).  These data  suggested  that  SIRT6  is a component of multiple large macromolecular complexes.  To purify such complexes and identify their components, we expressed epitope (Flag)-tagged SIRT6 protein in 293T cells, and carried out affinity-purification of Flag-SIRT6 complexes.  Coomassie stain of the Flag-immunoprecipititates (IPs) revealed several protein bands specific to the IP from the Flag-SIRT6-expressing cells compared to negative control cells (Figure 1b). SIRT6-specific bands were excised, and subjected to mass spectrometry.  This analysis identified DNA-PKcs, Ku80, and Ku70, which together comprise the DNA-PK holoenzyme, a central regulator of DNA DSB repair in mammalian cells (Figure 1b) [20-22].

The interaction of SIRT6 with DNA-PKcs, Ku80, and Ku70 was confirmed by Western analysis of the Flag-SIRT6 and negative control IPs (Figure 1c, d).  Notably, the interaction of SIRT6 with DNA-PKcs was resistant to ethidium bromide (Figure 1d), which disrupts DNA- dependent interactions.  In contrast, the association of Ku70 and Ku80 with SIRT6 was DNA-dependent (Figure 1d), similar to their interaction with DNA-PKcs [23,24].  These data suggest that SIRT6 interacts with the DNA-PK holoenzyme complex via the DNA-PKcs catalytic subunit.  Thus, we focused our analysis on the SIRT6-DNA-PKcs interaction. Using SIRT6-specific antibodies, we immunoprecipitated endogenous SIRT6 protein from 293T cells, and DNA-PKcs was specifically detected in these IPs (Figure 1e). Thus, our data indicate that SIRT6 and DNA-PKcs interact physically under physiologic conditions in cells.

### Mobilization of SIRT6 to chromatin in response to DNA damage

**Figure 2.**
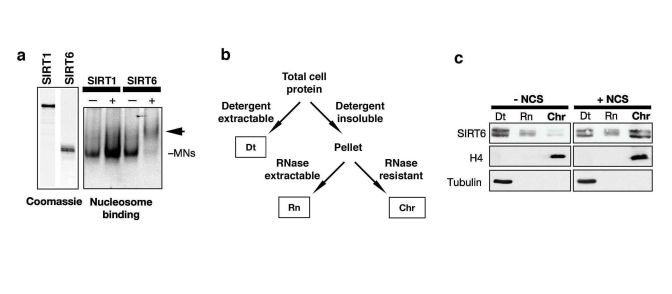
DNA damage stabilizes SIRT6 interaction with chromatin. (**a**) Gel shift showing SIRT6, but not SIRT1, binding to purified native nucleosomes in vitro.  Left: coomassie stain of recombinant SIRT6 and SIRT1 proteins.  Right: EtBr stain of nucleosomal DNA on a non-denaturing gel; arrow indicates SIRT6-mononucleosome complex.  (**b**) Schematic of chromatin purification protocol (see methods).  Dt, detergent extractable fraction; Rn, RNase extractable fraction; Chr, purified chromatin fraction.  (**c**) DNA damage-dependent stimulation of SIRT6 association with chromatin.  Western analysis with the indicated antibodies of HeLa cells treated with 45 nM neocarzinostatin (NCS) for 1 hour and fractionated as described in (**c**).  H4 and Tubulin are detected in the expected fractions.

In addition to the DNA-PK proteins, the purified SIRT6 complex also contained four low molecular weight bands with migration patterns characteristic of the core nucleosomal histones (Figure 1b). Western analysis of several different Flag-SIRT IPs confirmed that histones H2A, H2B, H3, and H4 are specifically associated with Flag-SIRT6 (data not shown). The observation that levels of the four core histones are similar in the SIRT6 IP (Figure 1b) suggested that the SIRT6-histone interactions could occur in the structural context of intact nucleosome particles. To investigate this possibility, we analyzed binding of purified, recombinant SIRT6 protein to purified mononucleosomes using an in vitro nucleosome binding assay.  SIRT6 protein bound efficiently to mononucleosomes, as manifested by a significant shift of the nucleosomes when fractionated on a non-denaturing gel; for comparison, SIRT1 did not show significant nucleosome binding in this assay (Figure 2a).  Together, these data provide evidence for a direct SIRT6 interaction with nucleosomes, the basic unit of chromatin.

The observation that SIRT6 interacts with a core DSB repair factor and with nucleosomes suggested that SIRT6 might modulate DNA DSB repair in the context of chromatin.  To investigate this possibility, we first asked whether the association of SIRT6 with chromatin might be dynamic and modulated by DNA damage. Previously, biochemical fractionation experiments showed that a majority of SIRT6 protein in cells is detected in an insoluble nuclear fraction enriched for chromatin [17].  To make it possible to detect potential changes in the strength of the SIRT6 interaction with chromatin, we used a more stringent chromatin fractionation protocol, under which, in the absence of DNA damage, only a minor portion of SIRT6 is associated with the purified chromatin fraction (Figure 2b, c) [25].  Upon exposure of cells to DNA damage, using the radiomimetic DNA DSB agent neocarzinostatin (NCS), this assay revealed a dramatic shift of SIRT6 protein into the chromatin-bound fraction (Figure 2c).  We conclude that DNA damage triggers increased association of SIRT6 with chromatin under physiologic conditions in cells.

### SIRT6-deficient cells show global hyperacetylation of H3K9 in response to DNA damage

**Figure 3.**
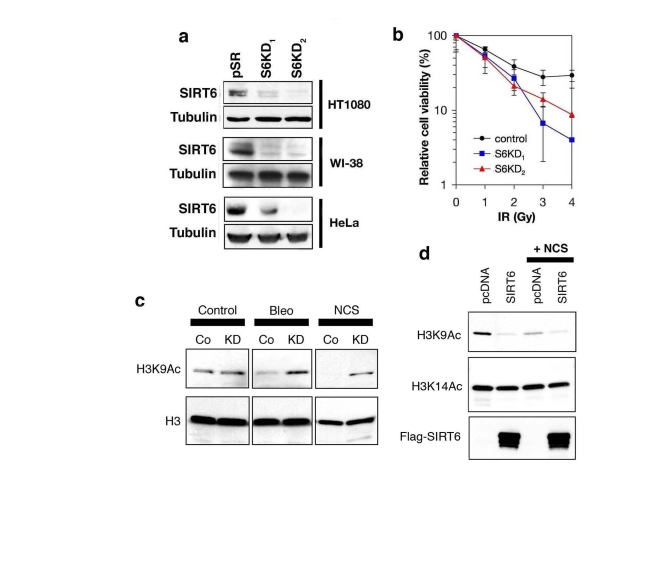
SIRT6 is required for global deacetylation of H3K9 in response to DNA damage. **(a)** Western analysis of SIRT6 expression levels in the indicated cell lines stably expressing two different SIRT6 shRNAs (S6KD1 and S6KD2) or empty vector control (pSR). **(b)** SIRT6 knock-down leads to hypersensitivity to γ-irradiation (IR).  Gy, Gray. **(c)** Western analysis of H3K9Ac levels in S6KD2 (KD) or control (Co) HT1080 cells in response to DNA DSB agents neocarzinostatin (NCS) or bleomycin (Bleo) treatment (1hr). **(d)** Western analysis of H3K9Ac levels in 293T cells over-expressing SIRT6 or empty vector control (pcDNA), following exposure to NCS (1hr).  In **(c)** and **(d)**, total H3 or H3K14Ac levels are shown as controls.

Because SIRT6 is a histone deacetylase with specificity for H3K9, we next asked whether alterations in cellular levels of SIRT6 protein might influence H3K9 acetylation (H3K9Ac) under DNA damage conditions. Therefore, we stably knocked down SIRT6 expression in cells by retroviral transduction of SIRT6-specific short-hairpin RNAs (shRNA) or negative control vector (pSR).  Two independent SIRT6 shRNAs (S6KD1 and S6KD2) resulted in significant knock-down (KD) of SIRT6 expression in multiple cell lines, as previously described (Figure 3a) [18]. We then confirmed that SIRT6 KD cells are hypersensitive to DNA damage (Figure 3b), demonstrating that the knockdown efficiency is sufficient to elicit known effects of SIRT6 inactivation [17].  Next, Western analysis was performed to compare levels of global H3K9 acetylation in SIRT6 KD or control pSR cells, under base-line conditions, or upon exposure to DNA DSB agents.  No difference in global H3K9Ac levels was observed in the absence of DNA damage (Figure 3c), consistent with our previous results [18].  However, when cells were treated with NCS or another DSB agent, bleomycin, H3K9Ac was substantially higher in SIRT6 KD cells compared to pSR cells (Figure 3c).  Interestingly, this effect corresponded to a DNA damage-dependent decrease in H3K9Ac in pSR cells, rather than a damage-dependent increase in H3K9Ac in the S6KD cells.  We also examined effects on H3K9Ac of over-expressing SIRT6 in the context of DNA damage.  SIRT6 over-expression led to a greater reduction in H3K9Ac levels than NCS treatment, and addition of NCS did not further decrease global H3K9Ac levels (Figure 3d). Together, our observations suggest that H3K9 is acutely deacetylated following treatment of cells with DNA damage, and SIRT6 is required for this process.

### SIRT6 is required for efficient mobilization of DNA-PKcs to chromatin following DNA damage

**Figure 4.**
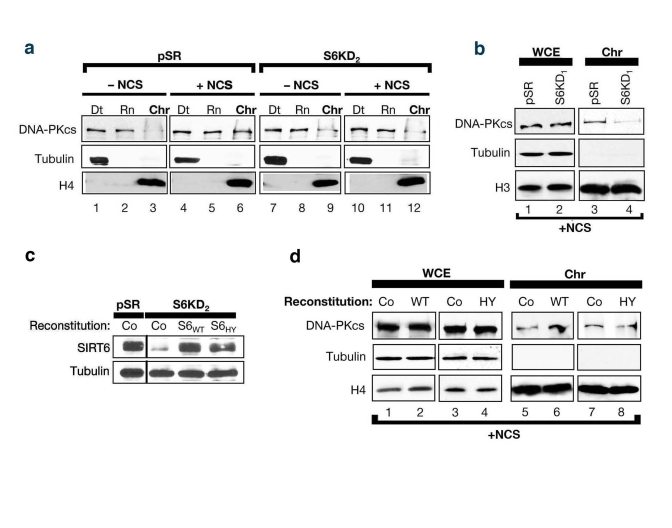
SIRT6 stabilizes DNA-PKcs at chromatin in response to DNA damage. **(a)** SIRT6 is required for efficient mobilization of DNA-PKcs to chromatin in response to the DNA DSB agent NCS.  Western analysis with the indicated antibodies of S6KD2 and control cells fractionated as in (Fig 2c).  **(b)** Fractionation experiments performed as in **(c)** utilizing S6KD1 cells (second independent SIRT6 shRNA). WCE, whole cell extract.  Chr, chromatin. **(b)** Western analysis showing reconstitution of S6KD2 cells with recombinant wild-type (WT) or catalytically mutant (HY) SIRT6 protein. **(d)** DNA-PKcs mobilization to chromatin upon NCS treatment was determined in S6KD2 cell lines reconstituted with WT SIRT6, catalytically inactive SIRT6 HY protein, or control vector (Co) as indicated.

Previous studies showed that exposure of cells to DNA DSB agents leads to mobilization of DNA-PKcs to chromatin [25].  To test whether SIRT6 might function in this context, the levels of chromatin-associated DNA-PKcs following exposure of SIRT6 KD and control cells to NCS were compared.  Consistent with previous studies [25], in the SIRT6-proficient pSR cells, DNA DSB generation was accompanied by an increase in the levels of DNA-PKcs at chromatin (Figure 4a, compare lane 6 to lane 3).  In contrast, no change in DNA-PKcs levels at chromatin was observed in SIRT6 KD cells in response to DNA damage (Figure 4a, lanes 9 and 12).  Similar results were observed in SIRT6 KD cells generated with the other SIRT6 shRNA (Figure 4b); thus, the changes in DNA-PKcs mobilization cannot be attributed to off-target shRNA effects.  Moreover, reconstitution of SIRT6 KD cells with wild-type SIRT6 protein (S6WT) restored DNA damage-dependent mobilization of DNA-PKcs to chromatin, whereas reconstitution with a catalytically inactive SIRT6 (S6HY) mutant protein did not (Figure 4c, d).  We conclude that functional SIRT6 protein is required for efficient stabilization of DNA-PKcs at chromatin in response to DNA damage.

### Dynamic association of SIRT6 and DNA-PKcs with chromatin flanking DNA DSBs

**Figure 5.**
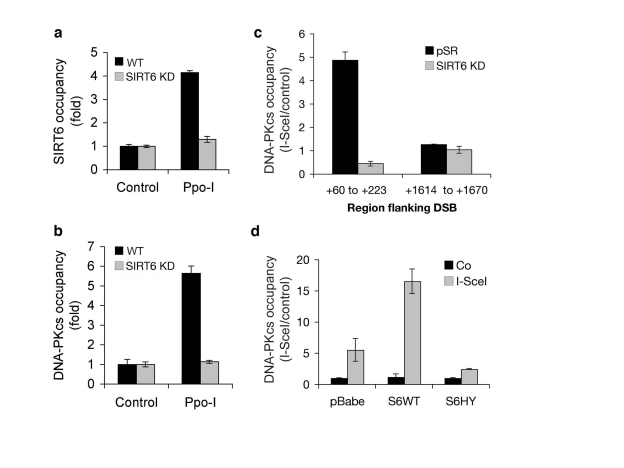
Dynamic association of SIRT6 and DNA-PKcs with chromatin flanking site-specific DNA DSBs. **(a)** SIRT6 occupancy at chromatin flanking DSBs induced by I-PpoI. Quantitative real-time PCR amplification of DNA sequences flanking the I-PpoI site from ChIPs performed with SIRT6 antibodies.  Data are normalized to no I-PpoI (control) samples.  **(b)** DNA-PKcs occupancy at chromatin flanking DSBs induced by I-PpoI, determined as for SIRT6 in **(a)**.  **(c)** DNA-PKcs occupancy at the indicated distances from an I-SceI DSB site in SIRT6 KD and controls cells.  Data are normalized to no I-SceI controls.  **(d)** DNA-PKcs occupancy at chromatin adjacent to (+60 to +223 bp) an I-SceI DSB site, following retroviral over-expression of Flag-tagged wild-type SIRT6 (S6WT), catalytically inactive SIRT6 (S6HY), or empty vector control (pBabe).  In all panels, SIRT6 KD cells were generated with S6KD2 shRNA, and the data represent the mean +/- S.E.

To study the dynamic movements of both SIRT6 and DNA-PK in the context of chromosomal DNA DSBs, we set up a recently described system to monitor molecular events that occur at chromatin adjacent to defined, endogenous DNA DSB target sites in human cells [26].  This system makes use of the eukaryotic homing endonuclease I-PpoI, which has ~300 target sites in the human genome [26].  To generate DSBs at these sites, we transduced human cell lines with a retroviral cassette containing an inducible system to express I-PpoI enzyme. Chromatin immunoprecipitation (ChIP) was then performed with SIRT6-specific antibodies, and the presence of chromatin near (182 bp) a specific DSB was determined by quantitative real-time PCR.  This analysis revealed that I-PpoI expression leads to a ~4-fold increase in mobilization of SIRT6 protein to chromatin adjacent to the DSB site (Figure 5a).  This increase in signal was abrogated in SIRT6 KD cells, confirming the specificity of the assay.

Next, we asked whether SIRT6 influences the recruitment of DNA-PKcs to the chromosomal DSBs.  Similar to SIRT6, DNA-PKcs was significantly increased (>5-fold) at sequences adjacent to the DSB following I-PpoI expression (Figure 5b).  Notably, depletion of SIRT6 in the SIRT6 KD cells dramatically reduced the mobilization of DNA-PKcs to the DSBs.  These results were confirmed with an independent site-specific break system, using the I-SceI endonuclease (Figure 5c) [27,28].  I-SceI has no endogenous target sites in human cells; therefore, we generated cells harboring stably integrated I-SceI target sites by retroviral transduction, and then expressed the I-SceI enzyme.  DNA DSB induction by I-SceI led to a ~5-fold increase in DNA-PKcs levels at sequences close to (~60-200 bp) the I-SceI target sequence, but not farther away (~1600-1670 bp) from the DSB (Figure 5c).  Again, this recruitment of DNA-PKcs was not observed in SIRT6 KD cells.

To determine whether increasing SIRT6 levels can further drive DNA-PKcs to DSBs, we also carried out the site-specific DSB analysis following retroviral over-expression of wild-type or catalytically inactive SIRT6 protein (Figure 5d). Over-expression of wild-type SIRT6 further increased DNA-PKcs at the DSB by ~3 fold.  In contrast, the mutant SIRT6-HY protein reduced the DNA-PKcs signal, suggesting a possible dominant-negative effect.  Together, our results demonstrate that SIRT6 is necessary and sufficient to promote efficient recruitment of DNA-PKcs to chromatin flanking site-specific DSBs.

### SIRT6 is required for efficient DNA DSB repair

**Figure 6.**
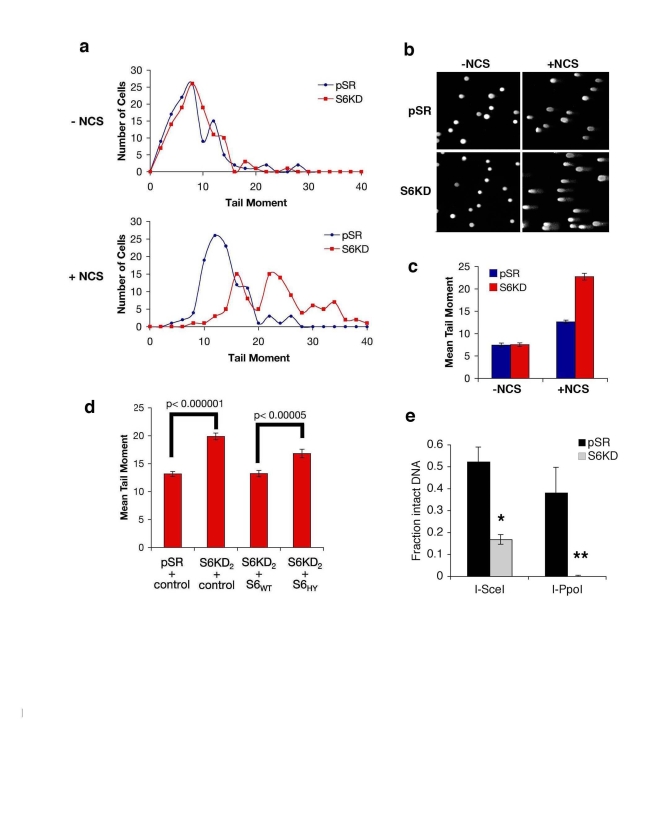
SIRT6 promotes resolution of DNA double strand breaks (DSBs). **(a-c)** Impaired resolution of DSBs in SIRT6 knock-down (S6KD2) cells.  Control (pSR) and knock-down (S6KD) cells were isolated 1 hour after NCS treatment and tail moment was determined in comet assays.  **(a)** Histogram of tail moments of >100 cells.  **(b)** Representative comet images from S6KD or control cells following DNA damage (+NCS) or mock (-NCS) treatment.  **(c)** Mean tail moment of comet assays shown in **(a)**.  Error bars indicate the S.E.M.  **(d)** Wild-type SIRT6 (S6WT), but not the mutant SIRT6 (S6HY), protein rescues the DSB repair defect of SIRT6 knock-down cells.  Mean tail moment of comet assays for the indicated cells, following treatment with NCS and quantified as in **(c)**.  **(e)** Resolution of site-specific DNA DSBs in SIRT6 KD (S6KD2) and control (pSR) cells assayed using the I-SceI and I-PpoI systems. Quantitative real-time PCR amplification of DNA using primers flanking the DSB sites is shown. *, p=0.009; **, p=0.02.  The data represent the average of triplicate experiments, and error bars indicate the S.E.M.

The above observations suggested that SIRT6 might influence the efficiency of DNA DSB repair.  To test this possibility, we used comet assays to compare levels of DNA damage in single-cells, at base-line conditions or upon exposure to DSB agents (Figure 6a). In this assay, cells were embedded in agarose plugs, and subjected to electrophoresis.  Intact genomic DNA shows very poor mobility under these conditions; DNA DSBs increase the migration of the DNA, generating "comets" of DNA detected by SYBR Green staining (Figure 6b).  The relative size of the comet "tail moment" was used to assess levels of DSBs in individual cells, and these values were plotted on a histogram for >100 cells (Figure 6a).  In the absence of NCS, the distribution of tail moments for SIRT6 KD and control cells were similar (Figure 6a, top).  In both cell types, NCS treatment led to a shift in tail moment distribution, with more cells showing larger amounts of DNA damage (Figure 6a, bottom).  However, this NCS-dependent increase in tail moment distribution was significantly greater for SIRT6 KD cells compared to control pSR cells.  This difference is reflected in an increased population Mean Tail Moment in NCS-treated SIRT6 KD cells (Figure 6c).  Together, these data provide evidence that SIRT6 KD leads to impaired resolution of DNA DSBs and increased accumulation of broken DNA.

To validate that the impaired chromatin mobilization of DNA-PKcs and increased DNA breaks observed in SIRT6 KD cells is specific to depletion of SIRT6, and to determine the role of SIRT6 enzymatic activity in this process, we used the S6KD cells reconstituted with the wild-type or catalytically inactive SIRT6 proteins (Figure 4c).  Complementation with wild-type SIRT6 protein in SIRT6 KD cells reduced the levels of NCS-dependent DSBs to levels observed in control cells (Figure 6d).  In contrast, the mutant SIRT6 protein was impaired in its ability to reverse the DNA DSB accumulation observed in SIRT6 KD cells  (Figure 6d).

**Figure 7.**
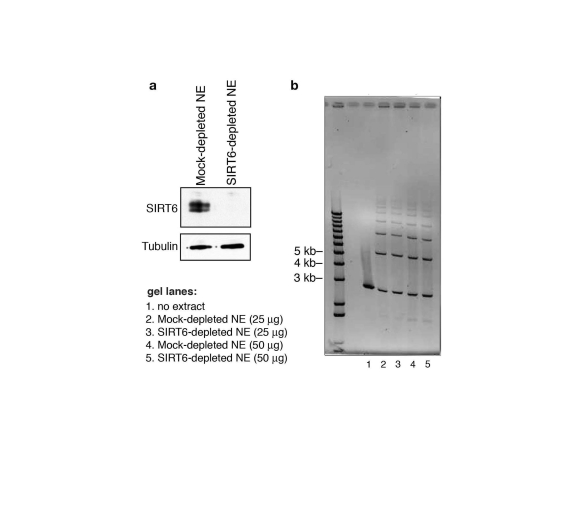
SIRT6 is not required for DNA double-strand break rejoining in a cell-free system. **(a)** Western analysis showing immunodepletion of SIRT6 from HeLa cell nuclear extracts (NE) with anti-SIRT6 antibodies. Mock-depleted control nuclear extracts were generated using the protein A/G-sepharose beads alone. **(b)** Ethidium bromide stain (inverted image) of DNA products of cell-free DSB rejoining assay.  Reactions contained linearized pUC19 plasmid DNA fragments and SIRT6- or mock- depleted nuclear extracts.  No difference in ligated species is observed between SIRT6-depleted and mock-depleted reactions.

Finally, to assay the effect of SIRT6 on DNA DSB repair by an independent method, we used the I-PpoI and I-SceI site-specific DSB systems.  Following DSB induction by expression of each endonuclease, PCR amplification of DNA was carried out using primers flanking the break sites. Comparison of the levels of PCR products was then used to quantify the relative efficiency with which the DSBs are resolved in SIRT6 KD or control cells.  In these assays, SIRT6 KD led to a ~4-fold decrease in the fraction of intact DNA present when assayed following I-SceI expression, and an even greater decrease using the I-PpoI system (Figure 6e). In contrast, SIRT6 depletion from nuclear extracts (Figure 7a) had no effect on DSB repair in an in vitro cell-free assay, in which the DNA is free and not bound within chromatin (Figure 7b). Thus, SIRT6 is important for the resolution of DNA DSBs, but only in the physiologic context of chromatin.  Together, these observations support a model in which SIRT6 impacts on DNA repair by modulating the association of DNA-PKcs at chromatin.

## Discussion

Previous work suggested that SIRT6 contributes in some way to DNA repair, because SIRT6-deficient mouse cells are hypersensitive to DNA damage and show increased genomic instability [17]; however, until now, direct evidence for SIRT6 function in DNA repair pathways has not been described.  In this study, we have now linked SIRT6 to DNA DSB repair, and show that SIRT6 modulates the levels of the DNA-PKcs DSB repair factor at chromatin surrounding DNA breaks.  It is possible that SIRT6 may also influence the chromatin association of other DNA repair factors.  For example, the spectrum of DNA damage agents to which SIRT6-deficient mouse cells are sensitive suggested a role for SIRT6 in Base Excision Repair (BER).  Thus, it will be interesting to investigate whether association of BER factors with chromatin at damaged DNA is regulated by SIRT6.

Our findings  in  this  study  are  somewhat unexpected, because previous results in SIRT6-deficient mice did not detect overt defects in Non-homologous End-joining (NHEJ), the specific DNA DSB repair pathway in which DNA-PK functions [17]. For example, SIRT6-deficient mice have normal lymphocyte development and V(D)J recombination, a process of programmed DNA recombination of immunoglobulin genes that depends on NHEJ activity [29].  We suggest that in the context of V(D)J recombination, the requirement for SIRT6 may be overridden by the action of lymphocyte-specific recombination factors, such as the RAG proteins, which are present at the sites of DSBs at V(D)J recombination substrates [21,29].  Repair of chromosomal DNA DSBs also appeared grossly normal in SIRT6-deficient mouse cells; it is possible that the assays for DNA DSBs used in our new study (comet assays for DNA damage in single cells and site-specific DSB repair assays) provide better sensitivity than the previously used assay (pulsed field gel electrophoresis) [17].  It is also possible that differences between the mouse study and our new findings in human cells may be due to compensatory mechanisms in the mouse knockouts.  Regardless, our study highlights the usefulness of complementing genetic studies in mice with biochemical analyses of human cell lines.

Regarding the molecular mechanism of SIRT6 action in response to DNA damage, our findings, that SIRT6 is required for stabilization of DNA-PKcs association with chromatin at DSBs and for global deacetylation of H3K9 upon DNA damage, are consistent with several potential models. For example, SIRT6 could be targeted to DSBs first and then recruit DNA-PKcs; DNA-PKcs could bind DSBs first and then recruit SIRT6; or the two proteins might associate with chromatin at DSBs cooperatively as a result of their protein-protein interaction.  The  fact  that  SIRT6  is  recruited  to  site-specific DSBs suggests a model in which it deacetylates H3K9 at chromatin surrounding the DSBs.  However, we were unable to detect reproducible effects of SIRT6 on H3K9Ac at DSBs using the site-specific DSB systems.  One possibility is that there is a tightly regulated acetylation/deacetylation cycle of H3K9 that occurs acutely in response to DNA damage, and effects of SIRT6 on this process could be beyond the resolution of our current assays.  SIRT6 might also impact on DNA damage-dependent H3K9Ac levels indirectly, for example, by altering the kinetics of cell-cycle dependent

histone acetylation fluctuations or through an as-yet undefined substrate.  Future studies of SIRT6 biology in these contexts should allow us to distinguish among these models.

### Perspective

In eukaryotic cells, genomic DNA is packaged in the higher order structure of chromatin, and regulation of chromatin plays a fundamental role in diverse epigenetic programs [30].  Such programs, in turn, can impact on aging-related molecular processes [31].  In this context, mammalian SIRT proteins that regulate histone deacetylation at chromatin can contribute to aging and life span regulation.  We recently showed that SIRT6 regulates both telomere function and aging-associated gene expression programs via site-specific deacetylation of H3K9 at chromatin, and loss of these activities in SIRT6-deficient cells contributes to premature senescence of human cells or a degenerative phenotype reminiscent of premature aging in mice [18,19].  Increasing evidence suggests that chromatin dynamics also play critical roles in the surveillance, detection, and repair of DNA damage [32,33].  The human genome is continually exposed to environmental and metabolic sources of DNA damage, the accumulation of which is proposed to contribute to genomic instability, aging, and age-related pathologies [31, 32-32].  Thus, chromatin regulatory factors that function in this context may be important aging modulators.  Recent work by Oberdoerffer et al showed that SIRT1 promotes repair of DSBs, and proposed that SIRT1 provides an example of how DNA damage-dependent redistribution of a chromatin modifying factor (the "RCM" response) may contribute to epigenetic changes that influence aging phenotypes [14].  Our findings regarding SIRT6 provide a second example  of  a  mammalian SIRT  functioning  in  DNA repair, and highlight the critical role of mammalian SIRT factors in linking chromatin regulation, DNA repair, and aging.

## Methods

Antibodies and constructs. SIRT6 antibodies were previously described [16].  Commercial antibodies: H3, H4, and Ku70 (Abcam); H2A, H2B, SIRT1, and a-Tubulin (Upstate); Flag (Sigma); GST, Ku80, and DNA-PKcs (Santa Cruz Biotechnology); DNA-PKcs (Neomarkers).  The Flag-tagged human SIRT1 expression construct was generated by sub-cloning into the p3xFlag vector (Sigma). Flag-tagged SIRT expression constructs in pcDNA were previously described [15]. Flag-SIRT6 wild-type and HY mutant retroviral expression constructs were generated by subcloning into the pBabe-puro vector.

Gel Filtration . HeLa cell nuclear extract was prepared as described previously [37].  3.5 mg of HeLa cell nuclear extract was loaded onto a 24-ml Superose 6 column (GE Healthcare) pre-equilibrated with 250 mM NaCl, 0.1% Triton X-100, 150 mM Tris pH 7.4, 10% glycerol buffer, run in the same buffer at a flow rate of 0.5 ml/minute.  72 fractions of 0.5 ml were collected and analyzed for the presence of SIRT6 by western blot.  Molecular weight standards (GE Healthcare) were used to calibrate the column as indicated.

Immunoprecipitation and purification of Flag-SIRT6 Complexes. 293T cells were transiently transfected using the TransIT-293 (Mirus) transfection reagent according to the manufacturer's instructions.  Cells were lysed 48 hrs post-transfection in IP-lysis buffer (50 mM Tris-HCl [pH 7.4], 250 mM NaCl, 0.25% Triton X-100, 10% glycerol, and complete protease inhibitor cocktail (Roche)).  The cell extracts were immuno-precipitated with anti-Flag M2 monoclonal antibody-conjugated agarose beads (Sigma).  Immunocomplexes were eluted with 3xFlag peptide (Sigma) and resolved by SDS-PAGE.  Proteins were analyzed by Western blot or Coomassie stained with Gelcode reagent (Pierce) and SIRT6-specific bands excised for analysis by mass spectrometry.  Where indicated, lysates were first incubated on ice for 30 minutes with 50 μg/ml ethidium bromide.

Nucleosome-binding assays. Mononucleosomes were purified from HeLa cells as described [38].  1.5 μg mononucleosomes were incubated with or without 10 μg of recombinant SIRT1 or SIRT6 protein for 30 minutes at 30°C in histone binding buffer (20 mM HEPES pH 7.9, 80 mM KCl, 0.1 mM ZnCl2, 0.1% EDTA, 10% glycerol, 0.1% NP40, 0.5 mM DTT, 1 mM PMSF) in a total volume of 20 μl. Reactions were fractionated on a 5% non-denaturing TBE gel, and mononucleosomal DNA stained with ethidium bromide.

Chromatin purification and cell fractionation. Cellular fractionation was performed as previously described [25].  Briefly, 1x106 HeLa cells were incubated  ± 760 ng/ml neocarzinostatin (NCS, Sigma) for 1 hour at 37°C, and washed and harvested in PBS.  The cell pellet was resuspended in 200 μl buffer 1 (150 mM NaCl, 50 mM Hepes 7.5, 1 mM EDTA, 0.1% Triton X-100, protease inhibitor cocktail (Roche) and phosphatase inhibitor cocktail (Sigma)) for 3 minutes on ice.  Lysates were pelleted at 13,000 rpm for 3 minutes, and the detergent extractable (Dt) supernatant collected.  The insoluble pellet was washed 2X in Buffer 1 without Triton X-100, resuspended in 100 μl Buffer 2 (150 mM NaCl, 50 mM Hepes 7.5, 1 mM EDTA, 200 μg/ml RNaseA, protease inhibitor cocktail (Roche) and phosphatase inhibitor cocktail (Sigma)), and incubated at 25°C for 30 minutes with gentle agitation.  Samples were centrifuged at 13,000 rpm for 3 minutes, and the RNase extractable (Rn) supernatant collected.  The remaining pellet (Rnase-resistant, purified chromatin sample (Chr)) was resuspended in SDS loading buffer, boiled, and sonicated for solubilization prior to Western analysis.

Generation of stable SIRT6 knock-down and reconstituted cell lines. SIRT6 knock-down cells (S6KD1 and S6KD2) were generated by retroviral transduction of shRNAs (target sequences: S6KD1, 5'- AAG CTG GAG CCC AAG GAG GAA-3'; S6KD2, 5'- AAG AAT GTG CCA AGT GTA AGA-3') in pSUPERretro (pSR), as previously described [18].  For reconstitution, S6WT and S6HY retroviruses were generated with pFBneo retroviral constructs as previously described [16].

DNA DSB and DNA damage sensitivity assays . IR sensitivity assays of SIRT6 KD and control WI-38 cells were performed using colony formation assays as previously described [17].  For detection of DSBs, SIRT6 KD and control HeLa cells were treated ±NCS (250 ng/ml, 30 minutes), and Comet assays performed according to the manufacturer's instructions (Trevigen).  Levels of DSBs, assessed via quantification of tail moments, were determined by CometScore software.  Approximately 100-125 cells were scored for each independent experiment.

Cell-free DNA DSB repair assay .  150 ng of purified  pUC19/HindII DNA fragments were incubated with 25 μg or 50 μg of SIRT6- or mock- depleted nuclear extracts for 1 hour at room temperature, proteins digested with proteinase K, and separated on a 0.7% agarose gel.  DNA species were visualized by ethidium bromide staining.

Mass Spectrometry . Gel slices were handled with a standard in-gel digest protocol (http://donatello.ucsf.edu/ingel.html). LCMSMS was run on a quadruple TOF instrument (QSTAR). Protein identification database search was carried out with MASCOT.

Site-specific DSB repair systems. The I-PpoI assays were performed as previously described [26].  Control and S6KD HT1080 cells were infected with the HA-ER-I-PpoI retrovirus for 24hrs.  The virus was removed and the cells were serum-starved for 36 hours in DMEM supplemented with 0.1% FBS for synchronization in G1.  The I-PpoI enzyme was then induced by the addition of 1uM 4-OHT (Sigma, St Louis, MO) for 24 hours.  The cells were harvested and genomic DNA from each sample was prepared using the Sigma GenElute mammalian genomic DNA mini-prep kit.  To analyze double strand break generation and repair, purified DNA was quantified and 50ug of DNA from each sample was used for Real-time PCR using primers that flank the I-Ppo1 site at chromosome 1 (5'-TCACTGAAGACTTGGTGGGA-3', 5'-AAACCATACGTGGCAGAGTG-3') and GAPDH (5'-GCTTGCCCTGTCCAGTTAAT3-', 5'-TAGCTCAGCTGCACCCTTTA3-') as a control.  For the I-SceI system, to generate HT1080 cells stably encoding the I-SceI target sequence (5'-ATTACCCTGTTATCCCTA-3') within a known sequence context (GFP cDNA), the I-SceI sequences were introduced into the MSCV retroviral transduction vector encoding GFP cDNA, and stably intergrated into the host genomic DNA by retroviral transduction.  To generate DSBs, I-SceI enzyme was expressed by transient transfection.  qRT-PCR primers for assaying intact DNA at the I-SceI target site are based on the known MSCV-GFP vector sequences flanking the I-SceI sequence: (5'-ACATGGTCCTGCTGGAGTTC-3') and (5'-TAAAGCGCATGCTCCAGACT-3').

Chromatin Immunoprecipitation (ChIP) at site-specific DSBs . ChIPs were performed as previously described [19]. Following endonuclease (I-SceI or PpoI) expression, DNA was cross-linked for 15 minutes with 1% formaldehyde and stopped in 0.125 M glycine.  Purified chromatin was sonicated to ~300 bp using the Bioruptor (Diagenode, Inc) and incubated with the indicated antibodies. Following reverse cross-linking, ChIP-associated sequences were detected by quantitative Real-Time PCR.  PCR primers: I-SceI +60 to +223, (5'-AGTCTGGAGCATGCGCTTTA-3') and (5'-GGGGAACTTCCTGACTAGGG-3'); I-SceI +1614 to +1670, (5'-CGCCTCAGCCAGCAACTC-3') and (5'-TAAGGCCGTTCTCTCGCATT-3'); I-PpoI, (5'-TTCACAGCACTCTCCATTCC-3' and 5'-TCTTTCCCACCAAGTCTTCA-3').
